# Effect of Modified Cow Dung Fibers on Strength and Autogenous Shrinkage of Alkali-Activated Slag Mortar

**DOI:** 10.3390/ma16206808

**Published:** 2023-10-22

**Authors:** Kang Li, Zhengxian Yang, Xueyuan Yan, Liying Xu, Bruno Briseghella, Giuseppe Carlo Marano

**Affiliations:** 1 Joint International Research Laboratory of Deterioration and Control of Coastal and Marine Infrastructures and Materials, College of Civil Engineering, Fuzhou University, Fuzhou 350108, China; 2 School of Civil Engineering and Architecture, Southwest University of Science and Technology, Mianyang 621010, China; 3 Department of Structural, Geotechnical and Building Engineering, Politecnico di Torino, Corso Duca degli Abruzzi 24, 10129 Torino, Italy

**Keywords:** cow dung fibers, alkali treatment, alkali-activated slag, ultrasonication, autogenous shrinkage

## Abstract

Alkali-activated slag (AAS) presents a promising alternative to ordinary Portland cement due to its cost effectiveness, environmental friendliness, and satisfactory durability characteristics. In this paper, cow dung waste was recycled as a renewable natural cellulose fiber, modified with alkali, and then added to AAS mortar. The physico-chemical characteristics of raw and modified cow dung fibers were determined through Fourier transform infrared (FTIR), X-ray diffraction (XRD), and Scanning electron microscope (SEM). Investigations were conducted on the dispersion of cow dung fibers in the AAS matrix, as well as the flowability, strength, and autogenous shrinkage of AAS mortar with varying cow dung fiber contents. The results indicated that modified fiber has higher crystallinity and surface roughness. The ultrasonic method showed superior effectiveness compared to pre-mixing and after-mixing methods. Compared with raw cow dung fibers, modified fibers led to an increase of 11.3% and 36.3% of the 28 d flexural strength and compressive strength of the AAS mortar, respectively. The modified cow dung fibers had a more significant inhibition on autogenous shrinkage, and the addition of 2 wt% cow dung fibers reduced the 7 d autogenous shrinkage of the AAS paste by 52.8% due to the “internal curing effect.” This study provides an alternative value-added recycling option for cow dung fibers as a potential environmentally friendly and sustainable reinforcing raw material for cementitious materials, which can be used to develop low autogenous shrinkage green composites.

## 1. Introduction

Ordinary Portland cement (OPC) as the main binder is widely used in the construction industry. However, the energy consumption and environmental pollution caused by OPC in the production process cannot be ignored. It has been reported that about 1.7 tonnes of raw materials (limestone, clay, etc.) are consumed and 0.73–0.85 tonnes of CO_2_ are released from the production of every tonne of OPC [1]. Therefore, the development of environmentally friendly and energy-saving alternatives to replace OPC has become a main research topic in the field of building materials. Alkali-activated materials have attracted enormous academic and industrial attention worldwide due to their remarkable mechanical properties and durability [2]. These materials can be prepared through the reaction of powdered aluminosilicate precursors (slag, fly ash, metakaolin, etc.) with aqueous solutions of alkaline activators (alkaline hydroxides, silicates, etc.) at room temperature or in a heated environment [3]. However, similar to Portland-cement-based products, alkali-activated materials also have the characteristics of high brittleness and low tensile strength. In addition, considerable shrinkage is also a key technical obstacle restricting the development of alkali-activated materials [4].

The incorporation of fibers into the alkali-activated matrix is a generally recognized approach to enhance the strengths and toughness of composites [5]. Bellum [6] found that steel fibers could be used to increase the flexural strength of fly-ash-slag based composites. Song et al. [7] reported that the addition of carbon fibers reduced the drying shrinkage of alkali-activated slag (AAS) concrete. Sun and Wu [8] claimed that 1% polyvinyl alcohol (PVA) fibers are the best content to increase the ductility of alkali-activated fly ash. However, most of these studied fibers have some issues. For instance, steel and carbon fibers are expensive, and the production of PVA requires phenols and amines as antioxidants and ultraviolet stabilizers, respectively, which is not beneficial to the sustainability [9]. Natural fibers have recently attracted wide attention because of their advantages of light weight, low cost, biodegradability, and regeneration [10,11]. Based on these characteristics, the research and utilization of natural fibers in alkali-activated materials are of great significance, especially in regions that lack fiber resources. Brakat and Zhang [12] indicated that the incorporation of natural cellulose fibers alleviated the autogenous shrinkage of AAS pastes by 35–67%. According to Wongsa et al. [13], the addition of sisal or coconut fibers to alkali-activated fly ash mortar enhanced the tensile and flexural strength of the mortar, similar to what glass fiber does. In addition, the mechanical properties are closely related to the dispersion of the fibers in the matrix. Ramezani et al. [14] pointed out that if the carbon nanotubes lack proper dispersion, the friction within the aggregated carbon nanotubes becomes minimized, and, as such, they tend to detach from the matrix, leading to reduction in the flexural strength of the composites. Many researchers have attempted to investigate various dispersion techniques to properly distribute the fibers in the cementitious matrix, including: pre-mixing, dry mixing, ball milling, and ultrasonication [15,16].

The accumulation of cow dung threatens the environment, and, to cope with this, the recycling of cow dung is becoming more diversified [17]. Recently, researchers have highlighted various applications of cow dung as a raw material, including its utilization in composting [18] and as a thermal insulator [19], adsorbent [20], and stabilizer [21]. Moreover, researchers have claimed that cow dung ash can be considered a cementitious material for preparing concrete. Zhou and Chen [22] found that the pozzolanic activity index of cow dung ash was higher than that of fly ash, indicating that cow dung ash was a suitable cement substitute for preparing concrete. Ramachandran et al. [23] used cow dung ash to partially replace 15% of OPC to prepare concrete. These insights indicate that using cow dung to prepare concrete could also bring great benefits, such as reducing the environmental impacts caused by the accumulation of cow dung and improving some key properties of concrete. However, there is currently little research that has focused on the application of cow dung fibers in construction materials, especially in alkali-activated materials. Therefore, the primary objective of this paper is to explore the feasibility of utilizing recycled cow dung fibers as a novel reinforcement fiber for producing AAS mortar. Considering the compatibility between the fibers and the matrix, the surface of the cow dung fibers was modified using an alkaline solution. Consequently, the physico-chemical characteristics of raw and modified cow dung fibers were determined through Fourier transform infrared (FTIR), X-ray diffraction (XRD), and Scanning electron microscope (SEM). To investigate the performance of AAS mortar reinforced with cow dung fibers, the fiber dispersion, flowability, mechanical properties, and autogenous shrinkage were also studied.

## 2. Experimental Section

### 2.1. Materials

**Figure 1:**
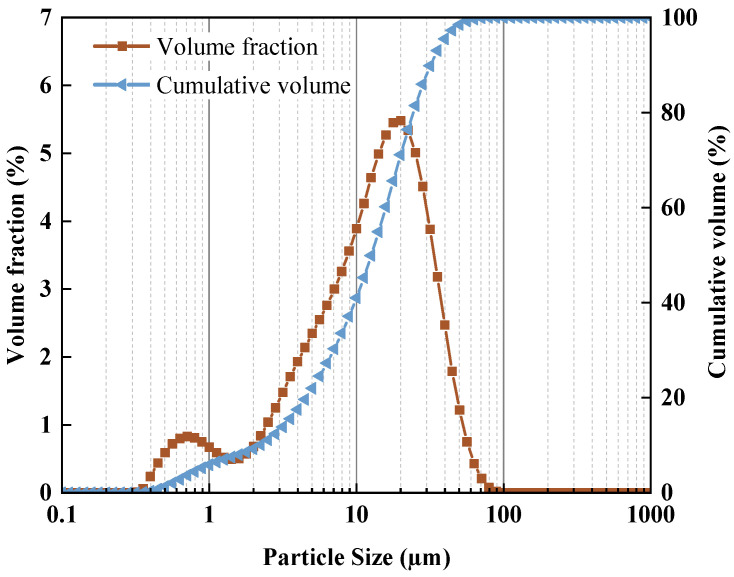
Particle size distribution of the slag.

**Figure 2:**
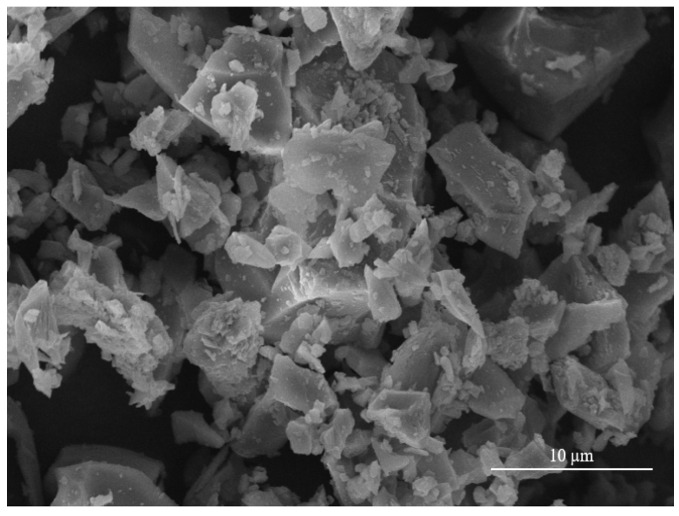
The SEM picture of the slag.

The precursor used in this study was S95-grade slag supplied by Longze Water Purification Material Co., Ltd., Gongyi, China. It had a specific surface area of 410 m^2^/kg and an apparent density of 2910 kg/m^3^. The chemical compositions of the slag are given in Table 1. The particle size distribution of the slag analyzed by laser granulometry is displayed in Figure 1, and Figure 2 shows an SEM picture of the slag. It can be observed that the slag consists of irregular and angular particles. The fine aggregate was standard sand with a fineness modulus of 2.48, purchased by Xiamen ISO Standard Sand Co., Ltd., Xiamen, China. Sodium hydroxide (NaOH, provided by Sinopharm Chemical Reagent Co., Ltd., Shanghai, China) of at least 96% purity was used as the activator. Polycarboxylate superplasticizer (PCS) used to adjust the flowability of designed mortars are obtained from Kezhijie New Material Group Co., Ltd., Xiamen, China.

**Figure 3:**
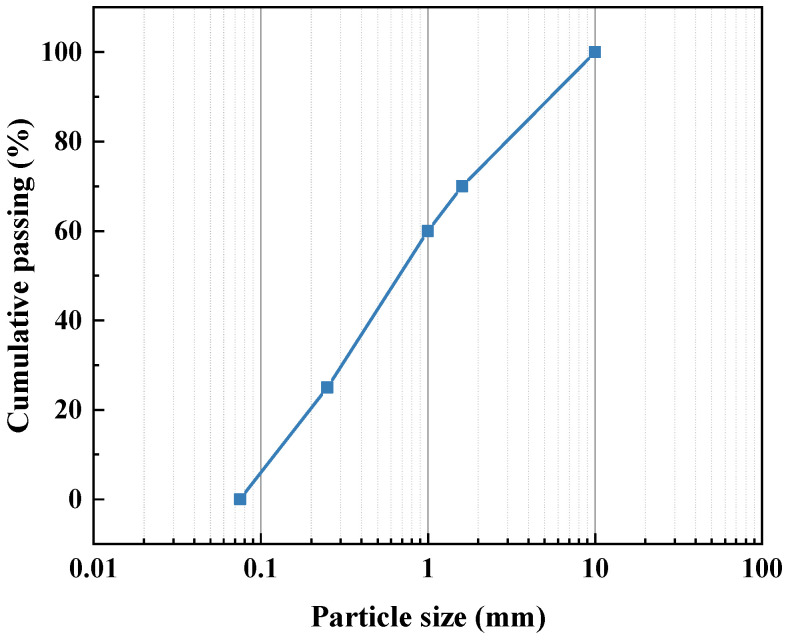
Particle size distribution of cow dung fibers.

The cow dung samples were obtained from a farm at Henan Agricultural University, China, where cows are primarily fed with corn stalks. Fresh cow dung samples were separated according to the method proposed by Li et al. [24]. During this process, the portion that does not pass through the sieve is collected and called cow dung fiber. The cow dung fiber was then dried at 60 °C until it reached a constant weight, and it was sealed up for use in later experiments. The properties of cow dung fibers are given in Table 2. The diameter of the fibers was measured using an optical digital microscope (Olympus DSX500, Tokyo, Japan), and the density of the fibers was determined using a specific gravity bottle. The tensile strength of the fibers has been previously documented in the work of Li et al. [24]. Figure 3 indicates the particle size distribution of cow dung fibers.

### 2.2. Modification of Cow Dung Fibers

Natural fibers have some limitations in reinforcing mortars. The strong alkaline environment in the matrix will cause the degradation of natural fibers and weaken the strength of fibers [25]. Furthermore, instability of the volume of the fibers weakens the fiber–matrix interface and leads to the development of microcracks while the composites are drying [26,27]. Surface modification of natural fibers becomes the key to preparing composites with excellent properties. Various modification techniques have been proposed in the literature, including alkali modification [28], acid modification [29], and silane modification [30]. Among the various literatures [31,32,33], alkali modification based on NaOH is favored because of the straightforward procedure, practicality, and affordability. Furthermore, this technique can improve the mechanical interlocking effect between the fiber and the matrix by increasing the surface roughness of the fiber [34]. In this research, cow dung fibers were immersed in a 1 mol/L NaOH solution for 12 h, after which they were repeatedly rinsed with deionized water to remove non-reacting alkali and dried for 12 h in an oven set to 60 °C.

### 2.3. Mortar Preparation

To assess the influence of fiber contents and modification on the performance of AAS mortar, several mortar mixtures reinforced with 0% (control), 0.5 wt%, 1 wt%, 1.5 wt%, and 2 wt% (by mass of slag) of raw or modified cow dung fiber were prepared. The mass ratio of slag to alkaline solution in each mixture was 0.5, while the mass ratio of slag to sand was 1:3. The mixture proportions are detailed in Table 3.

**Figure 4:**
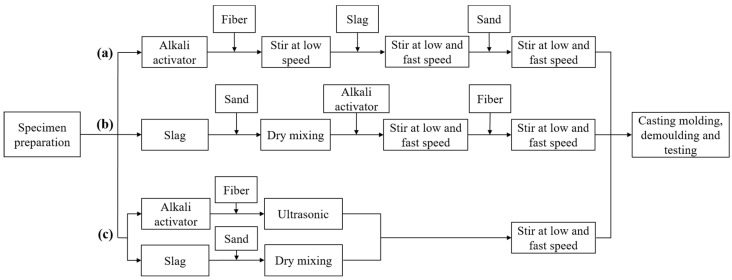
The preparation process of mixtures: (**a**) P_m_, (**b**) A_m_, and (**c**) U_m_.

The fibers need to be well-dispersed in the mortar to ensure their effectiveness in improving the matrix performance. The effects of the pre-mixing method (P_m_), after-mixing method (A_m_), and ultrasonic method (U_m_) on the dispersion of cow dung fiber in AAS mortars were considered. For the P_m_, cow dung fibers were initially combined with an alkali activator and subjected to low-speed stirring for 60 s. Subsequently, slag was introduced, and the mixture underwent 30 s of low-speed stirring followed by 30 s of fast stirring. Finally, sand was added, mixed at low speed for 30 s, and then rapidly stirred for an additional 30 s. For the A_m_, the initial step involved dry-mixing the slag with sand for 60 s. Following this, the alkali activator was introduced and subjected to 30 s of low-speed stirring and 30 s of fast stirring. Finally, the cow dung fibers were added and stirred for a total of 60 s, with 30 s each of low-speed and fast stirring. For the U_m_, cow dung fibers were added to an alkaline activator, and the mixture underwent ultrasonic treatment for 30 min. The binder material, along with standard sand, was dry-mixed for 120 s, and then the mixture containing cow dung fiber was gradually incorporated. The KQ-250 ultrasonic generator was used in this study. The ultrasonic frequency was 40 kHz, and the ultrasonic power was 250 W. The specific process is illustrated in Figure 4. All mortar samples were demolded after 1 day of curing at room temperature. These demolded samples were then moved to a fog room at a temperature of 20 ± 1 °C and a relative humidity of 95% for curing until the tests.

### 2.4. Testing and Analysis

#### 2.4.1. Fourier Transform Infrared (FTIR) Spectroscopy Analysis

The surface compositions of raw and modified cow dung fibers were determined using a Nicolet iS50 spectrometer (Thermo Scientific, Wilmington, MA, USA). The milled fiber samples were mixed with potassium bromide and compacted into a disc for analysis. The spectral range was 4000–500 cm^−1^ at a resolution of 2 cm^−1^.

#### 2.4.2. X-ray Diffraction (XRD) Analysis

The crystalline index (*CI*) of raw and alkali-modified cow dung fibers was ascertained through X-ray diffraction (X’pert3, Panalytical, Almelo, The Netherlands). The scanning range was from 5° to 80° 2θ at 40 kV and 40 mA. To provide a complete and even X-ray exposure, each specimen was leveled onto a brass-stub sample container in the form of powder. The *CI* of cow dung fibers was calculated based on the diffraction peaks of crystalline and amorphous matter proposed by Segal et al. [35] (Equation (1)).

(1) $$CI\;(\%)=\frac{I_{200}-I_{am}}{I_{200}}\times 100$$

where *I*_200_ represents the maximum intensity of diffraction of the (200) lattice peak at 2θ (22°–23°) and *I_am_* represents the minimum intensity of diffraction of the non-crystalline fraction, which is taken at 2θ (18°–19°).

#### 2.4.3. Water Absorption

The kinetics of water absorption behavior was studied by immersing raw and alkali-modified cow dung fibers in water for various amounts of time. The water absorption (*W*) was measured using the following Equation (2):

(2) $$W(\%)=\frac{M_{t}-M_{0}}{M_{0}}\times 100$$

where *M*_0_ is the dry mass of the fiber and *M_t_* is the mass of the fiber in air after immersion at time *t*.

#### 2.4.4. Dispersion

Fiber dispersion has an important effect on the properties of composites. If the fibers are not evenly dispersed within the concrete, it may affect the strength and shrinkage of the concrete [36]. Commonly used methods to evaluate fiber dispersion mainly include the fresh mixture method, the electrical resistance method, the simulation experiment method, and the ultrasonication energy index method [37,38]. Because the density of cow dung fibers used in this paper is lower than that of AAS mortar, which leads to different degrees of settling of cow dung fibers in fresh pastes, this paper adopts the fresh mixture method to evaluate the fiber dispersion in hardened specimens. To begin, samples were prepared using the process outlined in Figure 4, and then the hardened specimens were cut into 40 mm × 40 mm × 20 mm thin slices before finally being divided into 16 areas with a grid with an area of 10 mm × 10 mm. The fibers residing in each area were counted. According to the statistics of the 16 areas, the standard deviation (*S*), variation coefficient (*φ*), and dispersion coefficient (*β*) were calculated from Equations (3)–(5).

(3) $$S=\sqrt{\frac{\sum_{i=1}^{n}{\left(X_{i}-\bar{X}\right)}^{2}}{n-1}}$$

(4) $$\varphi =\frac{S}{\bar{X}}$$

(5) $$\beta =e^{-\varphi }$$

where *X_i_* is the number of fibers in area *i* of 16 areas; $\bar{X}$ is the average number of fibers in 16 areas; and *n* is the number of selected areas (*n* = 16 in this study). According to Equations (3)–(5), when the fiber distribution is ideally uniform, or the number of fibers in each area is equal, then *φ* = 0 and *β* = 1; if all fibers are only distributed in one area, then *φ* → ∞ and *β* → 0. In general, the *φ* is greater than 0, and the order of *β* is between 0 and 1. The smaller the *φ* or the higher the *β*, the better the dispersion of the fiber.

**Figure 5:**
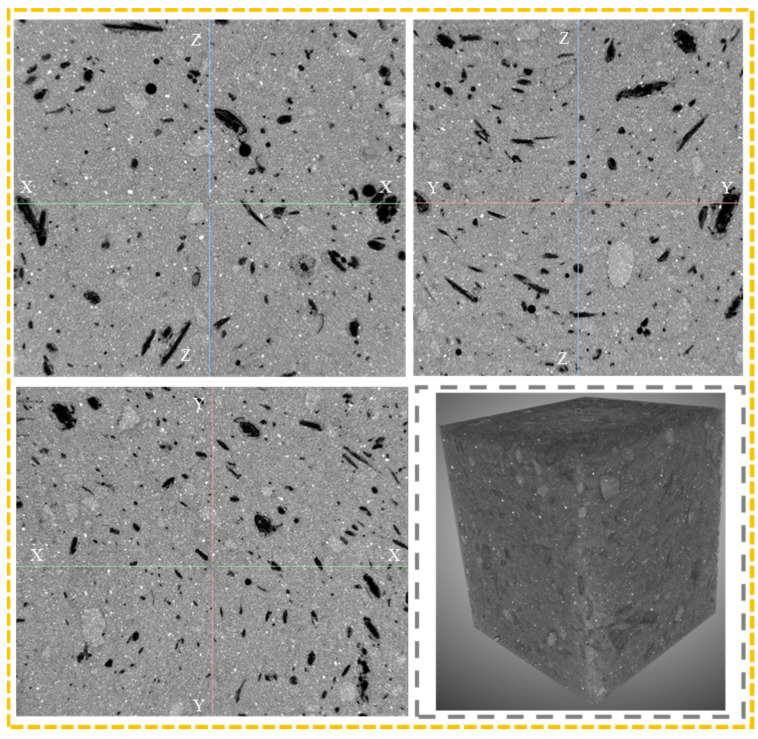
Segmentation of X-CT scanning images of cow dung fibers.

X-ray computed tomography (X-CT) (Zeiss, Jena, Germany) was used to further evaluate the dispersion of cow dung fiber in AAS mortar samples. Considering the similar pixel intensity of the fibers and the air voids, a more stringent filtration method based on the geometry of the fibers is required in order to separate the fibers from the air voids. In addition, cylindrical or rectangular cross-sections are considered optimal to minimize computer reconstructed image artifacts due to specimen inhomogeneity [39]. The image was segmented into slices on the x, y, and z axes, and the results are shown in Figure 5.

#### 2.4.5. Flowability

The workability of the AAS mortar mixtures was evaluated by flowability according to ASTM C1437-20 [40]. Before casting, a wet cloth was carefully used to wipe the flow table, truncated conical mold, tamper, and spatula. The freshly mixed mortar mixtures were subsequently added to the mold in two layers after mixing and rammed 20 times with a tamper to ensure that the mold filled evenly. A spatula was used to scrape the mortar off the top of the mold and wipe clean the empty area of the table. Then, the mold was gently lifted to make the flow table jolt 25 times in 15 s. A caliper was used to measure the diameter of two directions perpendicular to each other at the bottom of the mortar mixtures, and the average value was taken.

#### 2.4.6. Mechanical Properties

The mechanical properties of the AAS mortar were determined at 3, 14, and 28 days, especially its flexural and compressive strength. In accordance with ASTM C348-21 [41], sizes of 40 mm × 40 mm × 160 mm were fabricated to test the flexural strength, while the measurement of compressive strength testing was performed in conformity with ASTM C349-18 [42], and the samples were the broken halves of the prisms after the flexural test. The flexural and compressive tests were loaded at rates of 50 and 1400 N/s, respectively. Three parallel specimens were tested for each mixture.

#### 2.4.7. Autogenous Shrinkage

The autogenous shrinkage of the paste specimens was measured using corrugated tubes with a length of 425 ± 0.5 mm and a diameter of 29 ± 0.5 mm according to ASTM C1698-19 [43]. Fresh pastes were added to the corrugated tubes for the test, which involved placing them in a vibrating frame for two minutes to encourage the release of as many bubbles as possible. The temperature and relative humidity in the space were held constant at 23 ± 2 °C and 60 ± 2%, respectively. For 7 days, the data were logged every 15 min. After that, the autogenous shrinkage evolution slowed down, leading to the termination of the test. For each mixture, three replicates were tested.

#### 2.4.8. Morphological Analysis

The bonding capabilities of the cow dung fibers and the AAS matrix were studied using scanning electron microscopy (SEM, FEI Quanta 250, Hillsboro, OR, USA) in secondary electron (SE) mode; also, the microstructure of the AAS mortar was observed in backscattered scanning electron (BSE) mode to reveal the shrinkage mitigation mechanism of the cow dung fibers. The accelerating voltage applied was 25 kV. Before observation, the specimens needed to be covered with a layer of gold.

## 3. Results and Discussion

### 3.1. Modification of Cow Dung Fibers

**Figure 6:**
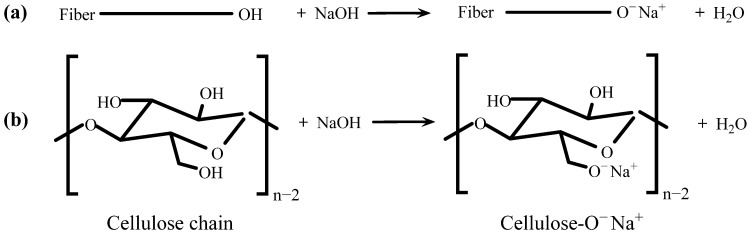
Reaction schemes of alkali modification: (**a**) fiber with NaOH; (**b**) cellulose with NaOH.

The hydroxyl groups of the fibers reacted with NaOH, as shown in Figure 6a,b. The results show that the free hydroxyl groups in cellulose, hemicellulose, and lignin could be decomposed by NaOH modification, and the water molecules could be released [34]. The schematic diagram of the reaction between cellulose and NaOH is presented in Figure 6b. This causes the rough surface of the fiber and removes the impurities and the amorphous parts of the fiber.

### 3.2. Chemical Characterization of Cow Dung Fibers

#### 3.2.1. Fourier Transform Infrared (FTIR) Spectroscopy Analysis

**Figure 7:**
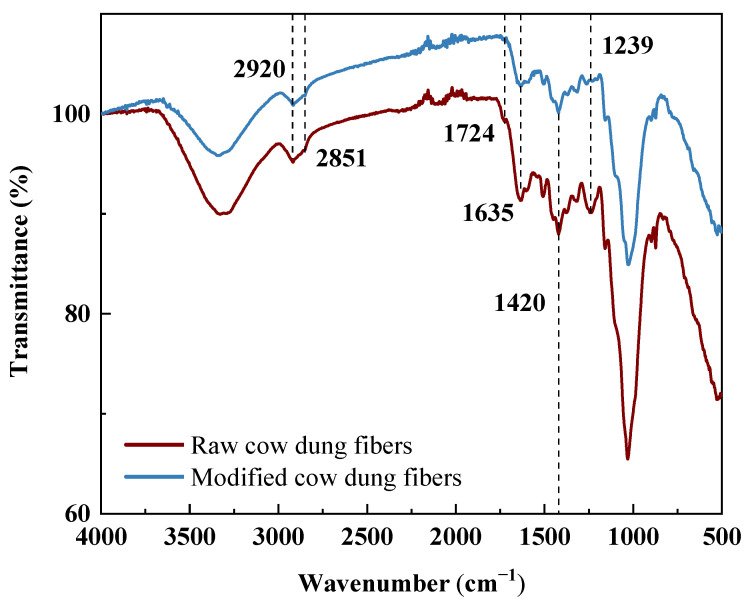
FTIR spectra of raw and modified cow dung fibers.

The FTIR spectra of raw and modified cow dung fibers are shown in Figure 7. Two main differences can be seen between raw and modified cow dung fibers. The peak at 1239 cm^−1^ corresponds to the C–O vibration of the acetyl group of lignin [24]. The peak almost disappeared after alkali modification, indicating that the NaOH solution could effectively remove the lignin from cow dung fibers. Secondly, compared with the raw cow dung fibers, there is no C=O stretching peak at 1724 cm^−1^ corresponding to the hemicellulose acetyl group in the modified cow dung fibers. This is due to the removal of hemicellulose from cow dung fibers after alkali modification. Interestingly, the significant peaks at 2850 and 2921 cm^−1^, which are caused by the stretching vibration of the C–H group, are mainly present in cellulose and hemicellulose [31]. Therefore, the results show that amorphous hemicellulose and lignin were removed through alkali modification. These results are in accordance with the observations reported in the literature [24,31].

#### 3.2.2. X-ray Diffraction (XRD) Analysis

**Figure 8:**
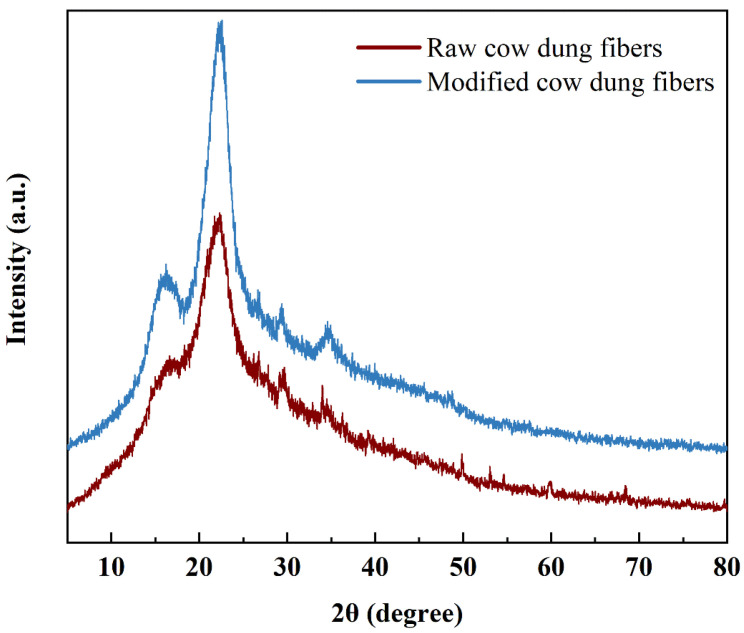
XRD patterns of raw and modified cow dung fibers.

Figure 8 shows the XRD patterns of the raw and modified cow dung fibers. It can be seen that the raw and modified cow dung fibers have similar patterns. The maximum peak representing crystalline macromolecules in the two types of fibers was obtained at 2θ = 22.40°, and the minimum peak representing amorphous macromolecules was obtained at 2θ = 18.24°. It can be inferred that cow dung fiber is a kind of semi-crystalline substance. The CI is crucial for characterizing crystalline cellulose because it determines the physico-chemical performance of the fiber [44]. A high CI normally indicates stiff and strong fibers. The CI can be calculated according to Equation (1), and the results are shown in Table 4. Compared with the raw cow dung fiber, the CI of the modified cow dung fiber is significantly increased by 37.7%. This is mainly because alkali modification removes some impurities, like lignin and hemicellulose. The crystalline regions of cellulose will be rearranged, and the cellulose chain will be better filled, resulting in higher crystallinity of the fiber [45,46].

### 3.3. Physical Characterization of Cow Dung Fibers

#### 3.3.1. Images of Cow Dung Fibers

**Figure 9:**
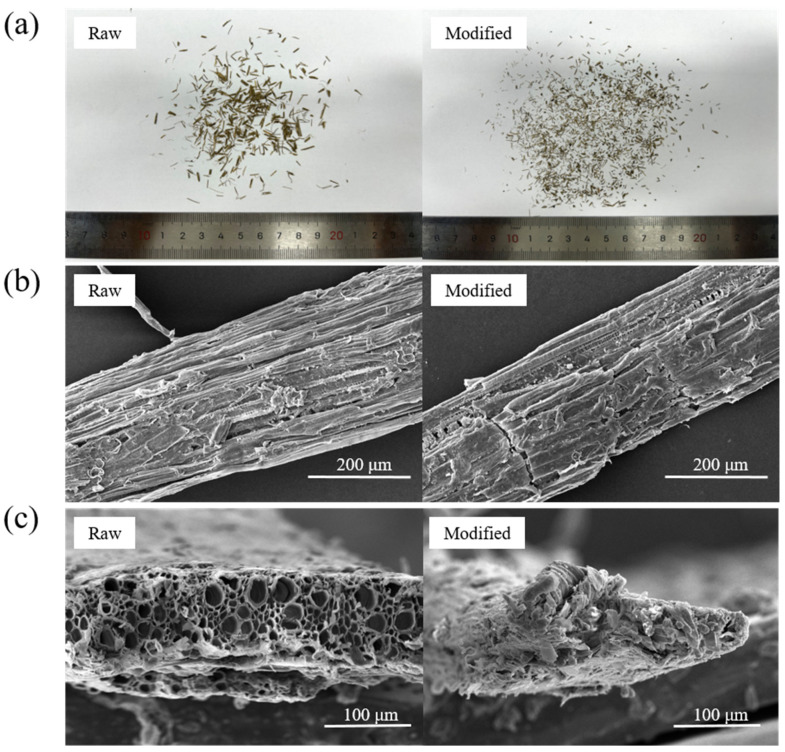
Images of raw and modified cow dung fibers: (**a**) appearance of cow dung fibers; (**b**) SEM images of fiber surface; (**c**) SEM images of the fiber end.

Figure 9 shows the appearance and SEM images of raw and modified cow dung fibers. It is observed from Figure 9a that after modification the fiber diameter decreased remarkably, due to the removal of amorphous components during modification. As can be seen from Figure 9b, the surface of the raw cow dung fiber was relatively smooth. However, the surface texture of the fiber becomes rough after modification, and more grooves can be observed. The rough surface of the fiber can enhance the mechanical interlocking, leading to greater interface bonding with the matrix. Figure 9c shows the end images of raw and modified cow dung fibers. The raw cow dung fibers can be seen to have a significant number of open lumens. These lumens allow the fiber to absorb a significant amount of water. However, the open lumen of modified cow dung fibers contracted or closed, and the microstructure was relatively dense. Accordingly, it can be speculated that the modification leads to a decrease in water absorption of the fiber. This speculation will be confirmed in the following water absorption test.

#### 3.3.2. Water Absorption of Cow Dung Fibers

**Figure 10:**
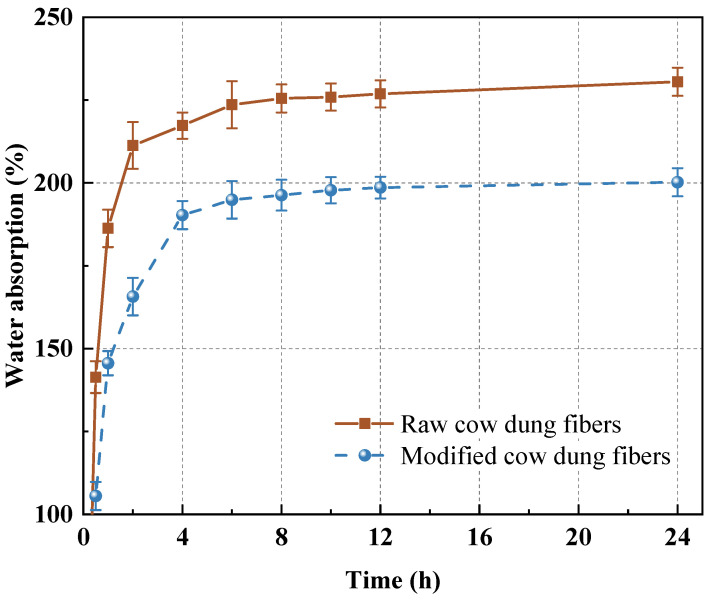
Water absorption of raw and modified cow dung fibers.

Figure 10 shows the water absorption rates of raw and modified cow dung fibers after being submerged in water for 0, 1, 2, 4, 6, 8, 10, 12, and 24 h. It is evident that as the immersion duration was prolonged, the cow dung fibers absorbed more water. The hydrophilic character is usually responsible for the chemical composition of the fiber (mainly cellulose and hemicellulose) and is therefore particularly sensitive to water [47,48]. As indicated in Figure 10, alkali modification reduces the water absorption of cow dung fibers because the alkali can break the hydrogen bonds between cellulose molecules. It is worth noting that high water absorption might have negative impacts on the cement matrix by lowering the effective water content and delaying cement hydration [31]. Thus, the adverse effects on the matrix can be alleviated through modification.

### 3.4. Dispersion of Cow Dung Fibers

Table 5 lists the dispersion parameters of cow dung fiber under different dispersion methods on the sample cross-section of AAS mortar. It can be observed that the fiber dispersion coefficient of all specimens is between 0.64 and 0.76. Moreover, the average dispersion coefficient of cow dung fibers is greater in the P_m_ (0.69) compared to the A_m_ (0.66) but lower than that in the U_m_ (0.75), indicating that the dispersion of cow dung fiber in AAS mortar after ultrasonic treatment is the best. The effect of ultrasonic treatment on the dispersion of cow dung fibers was attributed to the cavitation effect and acoustic flow effect produced by the ultrasonic waves in alkali-activated solutions containing cow dung fibers. These conditions greatly weaken the ability of cow dung fibers to clump together, effectively preventing the internal defects in the fibers from interacting with the mortar matrix to make them better dispersed.

**Figure 11:**
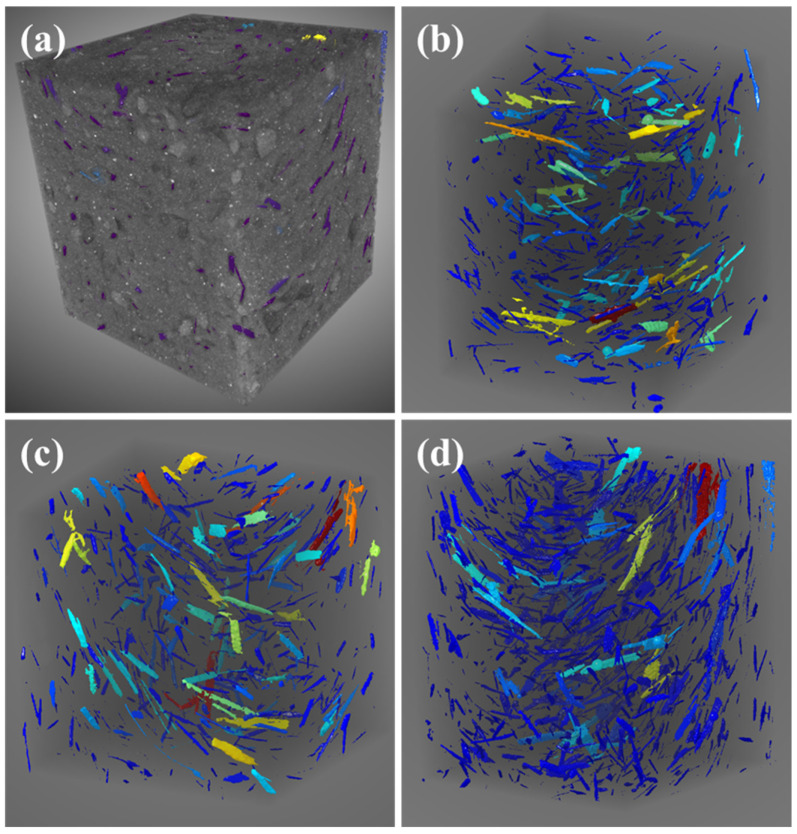
X-CT images of AAS mortar reinforced with cow dung fibers: (**a**) original 3D model; (**b**) P_m_; (**c**) A_m_; and (**d**) U_m_.

The X-CT images of cow-dung-fiber-reinforced AAS mortar under different dispersion methods are shown in Figure 11. It can be seen that the cow dung fibers mainly existed in the form of fiber bundles of different sizes, which overlapped with each other to form a complex three-dimensional network structure. Moreover, the best dispersion and random distribution of cow dung fibers in the AAS matrix after ultrasonication were all found, followed by the P_m_, and the worst dispersion was found in the A_m_. This finding is consistent with the above statistical results.

### 3.5. Flowability of AAS Mortar

**Figure 12:**
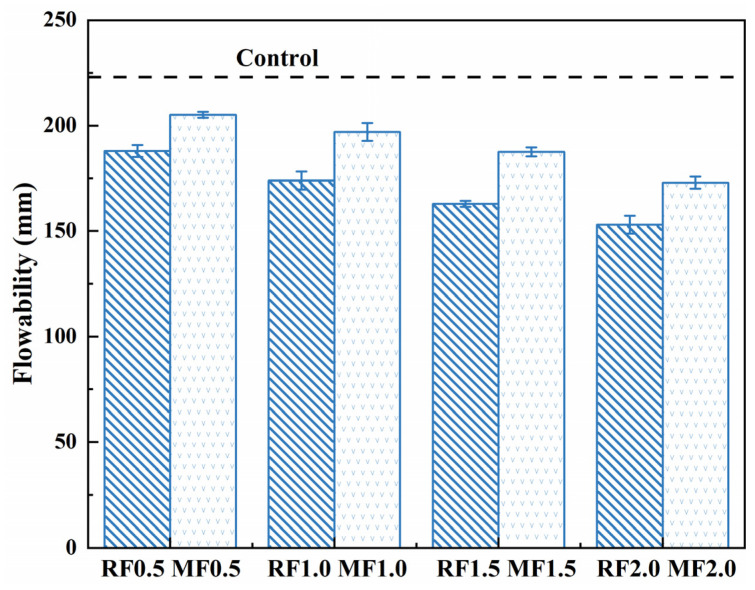
Flowabilities of AAS mortar mixtures.

Figure 12 presents the influence of raw and modified cow dung fibers incorporation on the flowability of AAS mortar mixtures. The control specimen without fibers had the highest flowability (spread of 223 mm). It is noticeable that the flowability of the mortar decreases with the addition of cow dung fiber, regardless of the fiber type (i.e., raw or modified). As expected, increasing the addition rate of cow dung fiber exacerbates the loss of flowability of the fresh mixture. This can be explained by two aspects [49]. First, cow dung fibers absorb free water due to their high porosity. Second, the higher dosage of cow dung fiber results in increasing the friction resistance to the flowability of AAS mortar mixtures due to its increased specific surface area. Moreover, the influence of raw fibers on the flowability reduction of the mixture seems to be slightly higher than that of the modified one. For instance, when the content of modified cow dung fiber is 0.5 wt%, 1 wt%, 1.5 wt%, and 2 wt% (by mass of slag), the flowability of the mixture is increased by 9.0%, 13.2%, 15.3%, and 13.8% compared with the raw cow dung fiber, respectively. In accordance with this observation, Rahimi et al. [50] indicated that the utilization of alkali-treated flax fiber improved the flowability of concrete.

### 3.6. Mechanical Properties of AAS Mortar

#### 3.6.1. Flexural Strength

**Figure 13:**
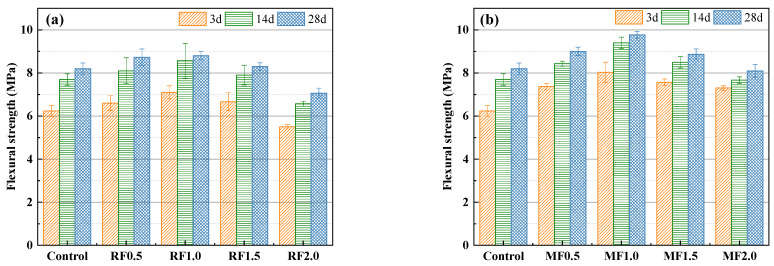
Flexural strengths of AAS mortars reinforced with (**a**) raw and (**b**) modified cow dung fibers.

Figure 13 shows the flexural strength of cow-dung-fiber-reinforced AAS mortar samples with various types and contents. It can be seen from Figure 13a that the flexural strengths of the mortars initially increased and subsequently decreased as the cow dung fiber content increased, reaching its maximum level at a content of 1 wt%. When 1 wt% of cow dung fiber was utilized, the flexural strength of the mortar on the 3rd, 14th, and 28th day increased by 14.5%, 11.7%, and 7.3% compared with that of the control group, respectively. This enhancement can be ascribed to the bridging effect given by the fibers. However, with a further increase in the cow dung fiber content from 1 wt% to 1.5 wt%, the fiber cannot be fully dispersed due to agglomeration in the matrix, which introduces more pores and defects [49]. The incorporation of 2 wt% cow dung fiber led to a slightly decreased flexural strength compared to the control mortar. This is because when the fiber exceeds a certain content, it cannot be completely wrapped by hydration products because the external load applied to the matrix cannot be effectively transferred to the fiber, which leads to the reduction in flexural strength.

The flexural strength of modified-cow-dung-fiber-reinforced AAS mortar is shown in Figure 13b. It can be observed that the change trend of the flexural strength of modified-fiber-reinforced AAS mortar is actually consistent with that of the raw fiber. However, the modified-fiber-reinforced AAS mortar specimens have higher strength than the raw-fiber-reinforced mortar specimens. For example, the flexural strengths of mortar samples prepared from 1 wt% of modified fiber at 3 days, 14 days, and 28 days were 8.0 MPa, 9.4 MPa, and 9.8 MPa, respectively, which were 12.7%, 9.3%, and 11.3% higher than those of the unmodified mortar samples. This phenomenon is connected to the effect of fiber modification. Alkali modification removes the amorphous parts on the outer surface of the fiber and produces a clean and rough surface, which improves the mechanical interlocking and interface adhesion between the fiber and the substrate, leading to improvement in the flexural strength of the modified-fiber-reinforced mortar. These results are consistent with previous findings from the literature [46].

#### 3.6.2. Compressive Strength

**Figure 14:**
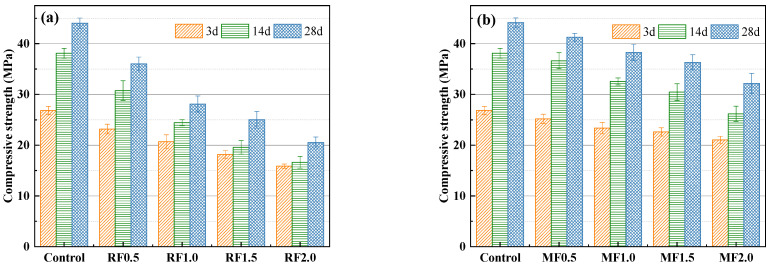
Compressive strengths of AAS mortars reinforced with (**a**) raw and (**b**) modified cow dung fibers.

The results of the compressive strength of AAS mortar specimens as a function of the fiber content at three test ages (i.e., 3 d, 14 d, and 28 d) are illustrated in Figure 14. With the increase in fiber content, the compressive strength of the mortar samples decreases. When the content of raw cow dung fiber was 0.5 wt%, 1 wt%, 1.5 wt%, and 2 wt%, compared with the control group, the compressive strength of the AAS mortar decreased by 13.8–23.7%, 22.8–40.0%, 32.1–48.6%, and 40.7–56.4%, respectively. The decrease in compressive strength can be associated with the increased porosity of fiber-reinforced AAS mortars [24,49]. The cohesiveness of the mortar samples weakens and their porosity increases as the fiber content increases, subsequently significantly decreasing the compressive strength of AAS mortar.

As shown from Figure 14a,b, the modified-fiber-reinforced mortars are stronger than the raw-fiber-reinforced mortars. Specifically, for mortar samples with 1 wt% fiber content, compared to the raw-fiber-reinforced mortar specimens, the modified-cow-dung-fiber-reinforced mortar presented compressive strength improvements of 13.0%, 33.6%, and 36.3%, respectively, with curing periods of 3, 14, and 28 days. This is because alkali modification removed the amorphous components (i.e., hemicellulose and lignin) in cow dung fiber and produced a rough surface, which improved the adhesion between the fiber and the matrix. This finding is consistent with the results obtained by Yan et al. [51] using modified-coir-fiber-reinforced concrete.

### 3.7. Autogenous Shrinkage of AAS Paste

**Figure 15:**
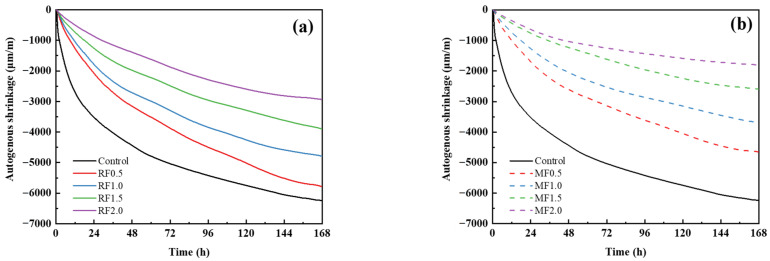
Autogenous shrinkage of AAS pastes reinforced with (**a**) raw and (**b**) modified cow dung fibers.

Figure 15 shows the measured autogenous shrinkage for all paste samples for a period of up to 7 days. As expected, the curves of each sample revealed the same trend, and the autogenous shrinkage of each group of paste mixtures gradually increased and tended to be stable with the increased time. It should be noted that the autogenous shrinkage of the control specimen develops fastest among all of the mixtures, reaching 3880 μm/m at 1 day and around 6240 μm/m at 7 days. The results are in line with previous research from Li et al. [52].

Additionally, Figure 15 demonstrates that when the fiber content increased, the autogenous shrinkage of all pastes reduced. At 7 days, when the raw cow dung fiber content was 0.5 wt%, 1 wt%, 1.5 wt%, and 2 wt%, the autogenous shrinkage of AAS pastes decreased by 7.3%, 23.2%, 37.7%, and 52.8%, respectively, compared with the control group. This is attributed to the fact that the cow dung fiber absorbs water and swells during mixing [53]. The consumption of water during the hydration of AAS leads to a decrease in the relative humidity inside the matrix. At this moment, the moisture initially absorbed by the fiber is released, which mitigates the occurrence of self-desiccation to a certain extent, i.e., the internal curing of the matrix [50]. Similar results have been reported for rice husk ash in AAS paste [54]. It should be noted that compared with the raw cow dung fiber, the modified cow dung fiber inhibited the autogenous shrinkage of paste specimens more significantly. For example, the autogenous shrinkage values at 7 days for the 1 wt% raw- and modified-cow-dung-fiber-reinforced paste sample was 4795 and 3680 μm/m, respectively. The first reason may be that raw cow dung fiber has a greater delay effect on the hydration of slag. Jo and Chakraborty [55] showed that organic components, such as hemicellulose and lignin, can surround the cement particles to form a protective film, which harms the crystal development of hydration products. Furthermore, the intense alkaline conditions of cement-based materials can also cause these organic components to break down into a significant number of sugars and carboxylic acids, further delaying the hydration of cement [56]. The second possible reason is that the curing efficiency of modified raw cow dung fiber becomes higher. The hemicellulose and lignin components were removed by alkali modification, resulting in increased cell wall porosity and altered water transport rates from the lumen and the cell wall to the surrounding AAS pastes [57]. Additionally, the surface texture of the modified cow dung fiber is rougher, resulting in enhanced adhesion between the fiber and the matrix, thereby increasing the pull-out resistance of the fiber from the matrix. As claimed by Kouta et al. [58], sisal fibers have the ability to bridge microcracks and prevent early-stage crack propagation, thereby reducing autogenous shrinkage.

**Figure 16:**
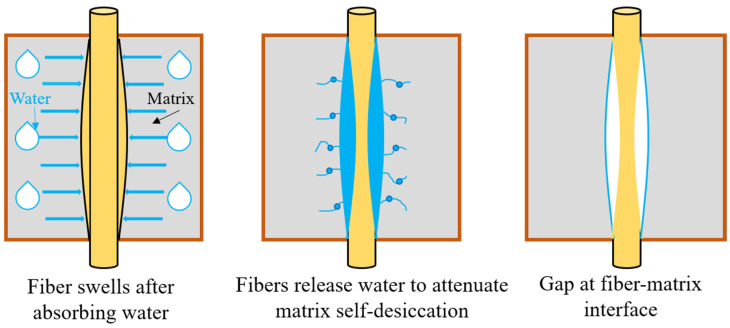
Schematic diagram of water absorption mechanism of cow dung fiber in AAS matrix.

**Figure 17:**
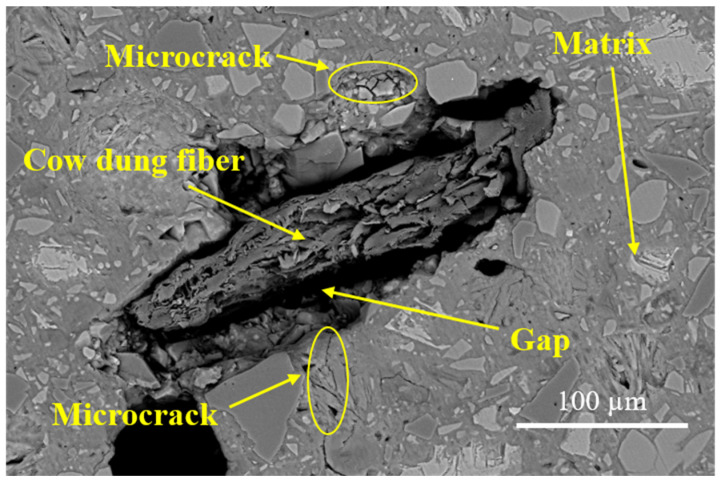
BSEM images of cow-dung-fiber-reinforced matrix of AAS mortar.

The whole water absorption process of cow dung fibers in the AAS matrix is shown in Figure 16. At first, the cow dung fiber swelled due to water absorption. With the continuous hydration of slag, the water inside the matrix was gradually consumed. At this time, the water initially absorbed by the fiber was released, effectively alleviating the occurrence of self-desiccation. As a result, a gap existed between the fiber and the matrix. Figure 17 shows the backscattered scanning electron microscopy (BSEM) images of cow-dung-fiber-reinforced AAS matrix at 7 days. It can be seen that there is a clear gap between the cow dung fiber and the matrix, which is attributed to the constriction of the cow dung fiber after compensating for the moisture inside the matrix. This explains the above autogenous shrinkage test results.

### 3.8. Morphological Analysis

**Figure 18:**
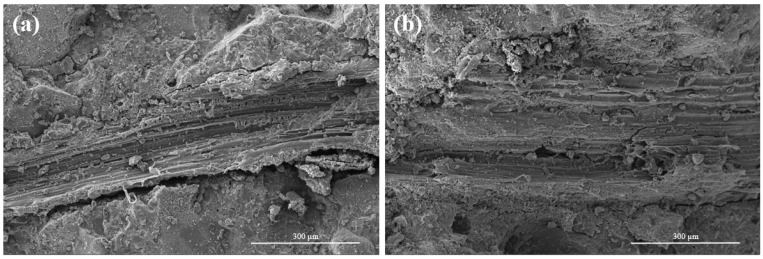
SEM images of AAS mortars reinforced with (**a**) raw and (**b**) modified cow dung fibers.

Figure 18 presents the SEM images of raw- and modified-cow-dung-fibers-reinforced AAS mortars at 28 days. It can be seen from Figure 18a that the interfacial transition zone between the fiber and the matrix is clearly visible in the raw-cow-dung-fiber-reinforced AAS mortar sample, indicating that the fiber–matrix interface adhesion is poor. Figure 18b shows that the surface roughness of cow dung fiber is improved through alkali modification, which promotes the fiber to be tightly surrounded by the matrix, and the fiber–matrix interface is well bonded. As a result, the strength of the AAS mortars reinforced with the addition of modified fibers increased in comparison to the raw fibers. These findings are consistent with the results of the mechanical properties.

## 4. Conclusions

In this paper, raw and modified cow dung fibers were used as reinforcement for AAS mortar. FTIR, XRD, and SEM investigations were carried out to examine the physico-chemical characteristics of raw and modified cow dung fibers. In addition to the dispersion of cow dung fibers in the AAS matrix, the effect of cow dung fibers on the flowability, strength, and autogenous shrinkage of AAS mortar was investigated. The following conclusions can be drawn:

(1) Alkali modification of cow dung fibers enhances cellulose crystallinity by dissolving amorphous components. This process also improves the roughness of the fiber surface and improves the bonding at the fiber–matrix interface.

(2) Ultrasonic dispersion is superior to pre-mixing and after-mixing. This can be attributed to the mechanical forces generated by ultrasonic treatment, as well as the acoustic flow effects it produces, leading to a uniformly dispersed fiber suspension.

(3) The incorporation of cow dung fibers results in a substantial improvement in the flexural strength of AAS mortars, which is mainly attributed to the bridging action between the mortar and the fibers. Additionally, after a curing period of 28 days, AAS mortars reinforced with 1 wt% modified cow dung fibers demonstrated a notable enhancement in both flexural and compressive strength, with increases of 11.3% and 36.3%, respectively, when compared to mortars containing untreated cow dung fibers.

(4) Incorporating cow dung fibers resulted in a significant decrease in autogenous shrinkage, mainly attributed to the “internal curing effect.” This effect occurred when the water absorbed by the cow dung fibers was released as the matrix’s internal humidity decreased, delaying self-desiccation. Furthermore, the alkali-modified cow dung fibers displayed an even more notable reduction in autogenous shrinkage due to their rough surface texture and the reduction in hemicellulose and lignin components.

## Figures and Tables

**Table 1:** Chemical compositions of the slag.

Materials	Chemical Composition (wt%)									
	CaO	SiO_2_	Al_2_O_3_	Fe_2_O_3_	MgO	Na_2_O	K_2_O	SO_3_	TiO_2_	LoI *
Slag	47.00	21.90	13.00	0.74	8.07	0.33	0.36	2.50	0.89	2.28

* LoI = Loss on ignition.

**Table 2:** Properties of cow dung fibers.

Properties	Cow Dung Fiber
Density (g/cm^3^)	1.37
Length (mm)	0.075–10
Average diameter (μm)	220.54
Tensile strength (MPa)	64.24

**Table 3:** Mix proportions of mortar with cow dung fiber *.

Sample	Slag (kg/m^3^)	Sand (kg/m^3^)	NaOH (kg/m^3^)	Water (kg/m^3^)	PCS (kg/m^3^)	Cow Dung Fiber	
						Content (% of Binder Weight)	Type
Control	558.1	1758	27.9	293	0.88	-	-
RF0.5	558.1	1758	27.9	293	0.88	0.5	Raw
RF1.0	558.1	1758	27.9	293	0.88	1.0	Raw
RF1.5	558.1	1758	27.9	293	0.88	1.5	Raw
RF2.0	558.1	1758	27.9	293	0.88	2.0	Raw
MF0.5	558.1	1758	27.9	293	0.88	0.5	Modified
MF1.0	558.1	1758	27.9	293	0.88	1.0	Modified
MF1.5	558.1	1758	27.9	293	0.88	1.5	Modified
MF2.0	558.1	1758	27.9	293	0.88	2.0	Modified

* RF0.5 represents the fiber-reinforced mortar with 0.5 wt% raw cow dung fiber content; MF0.5 represents the fiber-reinforced mortar with 0.5 wt% modified cow dung fiber content.

**Table 4:** Crystallinity index (CI) of raw and modified cow dung fibers.

Cow Dung Fibers	I_am_ (2θ = 18.24)	I_200_ (2θ = 22.40)	CI (%)
Raw	657	1240	47.0
Modified	623	1764	64.7

**Table 5:** Crystallinity index (CI) of raw and modified cow dung fibers *.

Number	$\bar{X}$	*S*	*φ*	*β*
P_m_1	7	2.66	0.38	0.68
P_m_2	7	2.65	0.38	0.68
P_m_3	6	2.18	0.36	0.70
A_m_1	7	2.80	0.40	0.67
A_m_2	7	3.13	0.45	0.64
A_m_3	7	2.89	0.41	0.66
U_m_1	7	2.13	0.30	0.74
U_m_2	7	1.92	0.27	0.76
U_m_3	7	1.95	0.28	0.76

* P_m_1 represents sample 1 prepared using the pre-mixing method; A_m_1 represents sample 1 prepared using the after-mixing method; U_m_1 represents sample 1 prepared using the ultrasonic method; and 1, 2, and 3 represent the serial number of the specimen (each group has three specimens).

## Data Availability

The data presented in this study are available upon request from the corresponding author.
